# Polymorphism discovery and association analyses of the interferon genes in type 1 diabetes

**DOI:** 10.1186/1471-2156-7-12

**Published:** 2006-02-22

**Authors:** Gerard AJ Morris, Christopher E Lowe, Jason D Cooper, Felicity Payne, Adrian Vella, Lisa Godfrey, John S Hulme, Neil M Walker, Barry C Healy, Alex C Lam, Paul A Lyons, John A Todd

**Affiliations:** 1 Juvenile Diabetes Research Foundation/Wellcome Trust Diabetes and Inflammation Laboratory, Cambridge Institute for Medical Research, University of Cambridge, Addenbrooke's Hospital, Hills Road, Cambridge, CB2 2XY, UK

## Abstract

**Background:**

The aetiology of the autoimmune disease type 1 diabetes (T1D) involves many genetic and environmental factors. Evidence suggests that innate immune responses, including the action of interferons, may also play a role in the initiation and/or pathogenic process of autoimmunity. In the present report, we have adopted a linkage disequilibrium (LD) mapping approach to test for an association between T1D and three regions encompassing 13 interferon alpha (IFNA) genes, interferon omega-1 (*IFNW1*), interferon beta-1 (*IFNB1*), interferon gamma (*IFNG*) and the interferon consensus-sequence binding protein 1 (*ICSBP1*).

**Results:**

We identified 238 variants, most, single nucleotide polymorphisms (SNPs), by sequencing *IFNA*, *IFNB1*, *IFNW1 *and *ICSBP1*, 98 of which where novel when compared to dbSNP build 124. We used polymorphisms identified in the SeattleSNP database for *INFG*. A set of tag SNPs was selected for each of the interferon and interferon-related genes to test for an association between T1D and this complex gene family. A total of 45 tag SNPs were selected and genotyped in a collection of 472 multiplex families.

**Conclusion:**

We have developed informative sets of SNPs for the interferon and interferon related genes. No statistical evidence of a major association between T1D and any of the interferon and interferon related genes tested was found.

## Background

Autoimmune diseases are often common chronic conditions that involve immune attack of one or more organ systems and affect approximately 5% of the population. Although the specific aetiologies of human autoimmune diseases remain largely unknown, in the case of type 1 diabetes [OMIM: 222100], four susceptibility loci have been identified and convincingly replicated: the HLA class II genes on chromosome 6p21[1], the insulin gene on chromosome 11p15[2,3], the CTLA-4 gene on chromosome 2q33[4,5], and the PTPN22 gene on chromosome 1p13[6,7]. Evidence for a fifth gene has recently been reported, *IL2RA *(*CD25*), encoding the α-subunit of the IL-2 receptor on chromosome 10p15[8]. Here, we have adopted a linkage disequilibrium mapping approach to test for an association between T1D and three regions encompassing 13 IFNA genes (*IFNA1 *[OMIM: 147660], *IFNA2 *[OMIM: 147562], *IFNA4 *[OMIM: 147564], *IFNA5 *[OMIM: 147565], *IFNA6 *[OMIM: 147566], *IFNA7 *[OMIM: 147567], *IFNA8 *[OMIM: 147568], *IFNA10 *[OMIM: 147577], *IFNA13 *[OMIM: 147578], *IFNA14 *[OMIM: 147579], *IFNA16 *[OMIM: 147580], *IFNA17 *[OMIM: 147583] and *IFNA21 *[OMIM: 147584]), *IFNW1 *[OMIM: 147553], *IFNB1 *[OMIM: 147640], *IFNG *[OMIM: 147570] and *ICSBP1 *[OMIM: 601565], using tag SNPs [9-11] in a collection of 472 multiplex families. We have previously shown that the tag SNP approach can reduce genotyping costs by approximately two-thirds [10-12].

The type I interferons, including the IFNAs, IFNB1 and IFNW1, are a large, evolutionarily-conserved family of homologous pro-inflammatory antiviral, immune-regulatory, cytokines, encoded by a cluster of single exon genes in a 400 kb region of human chromosome 9p21.3, and the orthologous ~400 kb region of mouse chromosome 4. The type II interferon, IFNG, encoded by a four-exon gene on chromosome 12, also exhibits antiviral activity but in contrast to the type I interferons, its main biological activity appears to be immunomodulatory. Type I interferons have increased prior probability in terms of being associated with susceptibility to human immune-mediated disease because this region has been linked with susceptibility to a number of mouse models of autoimmune diseases and related traits [13-16], although, to date, there is no evidence of linkage in humans.

We also assessed the related *ICSBP1*, the product of which, a transcription factor of the interferon regulatory factor (IRF) family, plays a major role in interferon signalling. Although nine distinct IRFs have been described[17], we analysed *ICSBP1*, specifically because chromosome 16q24.1, the region containing the nine-exon gene encoding ICSBP1, has shown some evidence of linkage to T1D previously [18-20].

## Results

### IFNA gene cluster tag SNP analysis

The resequencing of 13 IFNA genes in 32 T1D cases identified 152 polymorphisms (see Additional file 1), 144 of which were SNPs and eight were deletion/insertion polymorphisms (DIPs); of these, 64 SNPs and eight DIPs were novel when compared with dbSNP build 124. Thirty coding SNPs were identified in nine out of the 13 IFNA genes, of which six were synonymous, and 24 were non-synonymous, including the previously known premature stop codon polymorphism[21,22], in the IFNA10 gene, at predicted amino acid residue position 20 [Cys-Stop]. Seventy-five polymorphisms had a minor allele frequency (MAF) < 0.1 and were consequently not included in the tag SNP selection. As the LD within and between the 13 IFNA genes is strong, a set of tag SNPs was selected for the region encompassing the 13 IFNA genes. From the 77 polymorphisms (MAF ≥ 0.1), 20 tag SNPs were selected (minimum *R*^2 ^= 0.81) and genotyped in the family collection. All tag SNP genotypes in parents and T1D affected offspring were in Hardy-Weinberg equilibrium (HWE). The multilocus test[10,11]*P*-value was 0.35 (1,335 parent-child trios, χ_20_^2 ^= 21.9; see Additional file 2).

### *IFNB1 *tag SNP analysis

The resequencing of *IFNB1 *in 32 T1D cases identified 21 polymorphisms (including one synonymous SNP), 18 of which were SNPs and three were DIPs; of these, five SNPs and three DIPs were novel (see Additional file 3). Ten polymorphisms had a MAF < 0.05 and were consequently not included in the tag SNP selection. From the 11 polymorphisms (MAF ≥ 0.05), four tag SNPs were selected (minimum *R*^2 ^= 0.83) and genotyped in the family collection. All tag SNP genotypes in parents and T1D affected offspring were in HWE, except for rs10811465, which deviated from HWE in the parents (*P *= 0.0088; excess homozygotes). As there were no apparent errors with the genotype scoring, this SNP was re-typed using an alternative Taqman assay to check for a genotyping error. We found a high correlation of genotypes between the two assays (correlation coefficient = 0.99) and consequently, that the SNP genotypes still deviated from HWE in parents (*P *= 0.0012; excess homozygotes). Blast searches of the primer and probe sequences suggested that the assays should be specific, reducing the likelihood of deviation due to gene duplication. As the multilocus test does not assume HWE[8], we proceeded to analyse the set of tag SNPs. The multilocus test *P*-value was 0.12 (1,427 trios, χ_4_^2 ^= 7.2; see Additional file 4).

### *IFNG *tag SNP and single SNP analysis

The SeattleSNP variation discovery resource (http://pga.gs.washington.edu/) in 23 European Americans identified 13 polymorphisms in their resequencing of *IFNG*, 12 of which were SNPs and one was a DIP. Six polymorphisms had a MAF < 0.1 and were consequently not included in the tag SNP selection[23]. From the seven polymorpisms (MAF ≥ 0.1), four tag SNPs were selected (minimum *R*^2 ^= 0.84) and genotyped in the family collection (see Additional file 5). All tag SNP genotypes in parents and T1D affected offspring were in HWE. The multilocus test *P*-value was 0.43 (1,417 trios, χ_4_^2 ^= 3.8; see Additional file 6).

An additional *IFNG *SNP was obtained from the literature, rs2430561[24], with a reported association with tuberculosis. This common SNP (MAF = 0.46) was genotyped in the family collection. SNP genotypes in parents and T1D affected offspring were in HWE. The transmission/disequilibrium test[25]*P-*value was 0.091 (1,157 trios, χ_1_^2 ^= 2.9; see Additional file 6).

### *IFNW1 *tag SNP analysis

The resequencing of *IFNW1 *in 32 T1D cases identified 23 polymorphisms (see Additional file 7), of which 21 were SNPs and two were DIPs; of these, seven SNPs and two DIPs were novel. One SNP had a MAF < 0.05 and was consequently not included in the tag SNP selection. From the 22 polymorphisms (MAF ≥ 0.05), 10 tag SNPs were selected (minimum *R*^2 ^= 0.88) and genotyped in the family collection. All tag SNP genotypes in parents and T1D affected offspring were in HWE, except for rs12554686, which deviated from HWE in the parents (*P *= 0.0019; fewer homozygotes than expected under HWE). As there appear to be no obvious errors with the original Invader genotype scoring, this SNP was also re-typed using an alternative Taqman assay to check for a genotyping error. We found a high correlation of genotypes between the two assays (correlation coefficient = 0.96) and consequently, that the SNP genotypes still deviated from HWE in parents (*P *= 0.0024; fewer homozygotes). As with rs10811465 in *IFNB1*, blast searches suggested that the assays should be specific, reducing the likelihood of deviation due to gene duplication. The multilocus test *P*-value was 0.90 (1,401 trios, χ_10_^2 ^= 4.9; see Additional file 8).

### *ICSBP1 *tag SNP analysis

The resequencing of *ICSBP1 *in 32 T1D cases identified 42 polymorphisms, including one non-synonymous and four synonymous SNPs in exon seven of the gene (see Additional file 9). Forty of the 42 polymorphisms identified were SNPs and two were DIPs; eight of these SNPs and one DIP were novel. Eleven polymorphisms had a MAF < 0.05 and were consequently not included in the tag SNP selection. From the 31 polymorphisms (MAF ≥ 0.05), seven tag SNPs were selected (minimum *R*^2 ^= 0.82) and genotyped in the family collection. All tag SNP genotypes in parents and T1D affected offspring were in HWE. The multilocus test *P*-value was 0.58 (1,411 trios, χ_7_^2 ^= 5.6; see Additional file 10).

## Conclusion

As we found no statistical evidence of an association between T1D and any of the interferon and interferon related genes tested (Table 1). We conclude that the IFNAs, IFNB1, IFNW1, ICSBP1 and IFNG genes do not contribute significantly to T1D in the populations analysed. Of course, it remains possible that there exists a common disease variant in any of these genes, which either has an effect smaller than would be detected with this study size or is in much weaker LD with the tag SNPs than any other polymorphism known to us[12]. However, had we genotyped all the common polymorphisms (45 tags selected from 148 common polymorphisms), we would have been little better able to detect such a variant. The LD mapping approach has provided a cost-effective T1D association study of this complex gene family and, in addition, a better quality polymorphism map for others to use in the genetic analyses of other diseases.

**Table 1:** Summary of disease association results. Number of individuals in the sequencing panel, number of polymorphisms identified per gene, number of tag SNPs selected. Minor allele frequency cut-off used in tag SNP selection, minimum R^2 ^of each set of tag SNPs, number of trios analysed, χ^2 ^results and multilocus test P values for the IFNA gene cluster, IFNB1, IFNW1 and IFNG.

		Number of						
SNP/Gene/Region	Individuals sequenced	Polymorphisms	Tag SNPs	MAF cut-off	Min *R*^2^	Trios	χ^2^	P

*IFNA*	32	77	20	0.1	0.81	1,335	21.9	0.35
*IFNB1*	32	11	4	0.05	0.83	1,427	7.2	0.12
*IFNW1*	32	22	10	0.05	0.88	1,401	4.9	0.90
*IFNG*	23	7	4	0.1	0.84	1,417	3.8	0.43
*IFNG *– rs2430561	-	-	-	-	-	1,157	2.9	0.091

## Methods

### Polymorphism discovery

The genes of interest, with the exception of *IFNG*, were annotated locally[26,27] and displayed through gbrowse[28] within T1DBase[29]. Using these annotations, polymorphisms were identified by resequencing, using a nested PCR approach, the exons, exon/intron boundaries and up to 3 kb of 5' and 3' flanking sequence, in DNA samples from 32 T1D patients. The sequencing reactions were performed using Applied Biosystems (ABI) BigDye terminator chemistry and the sequences were resolved using an ABI 3700 DNA Analyser. Sequence traces were analysed using the Staden package[30] and double-scored by a second operator. In the case of INFG, polymorphisms identified from 23 individuals of European descent were extracted from the SeattleSNP database[31].

### Tag SNP selection

**Figure 1:**
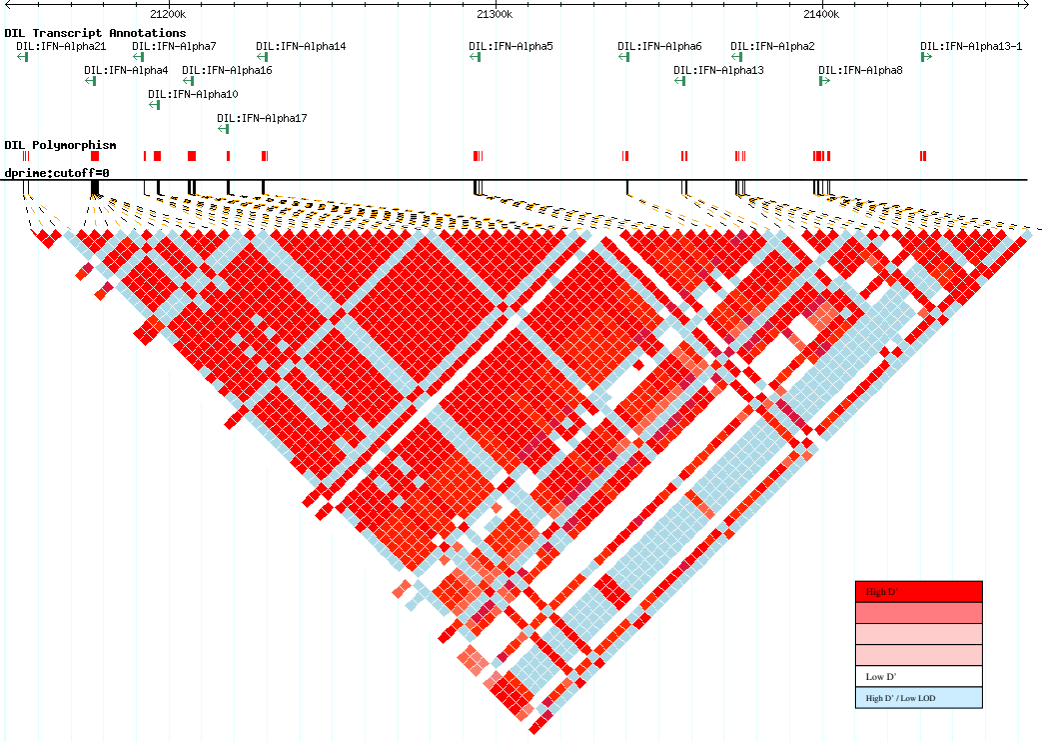
**Linkage disequilibrium across the IFNA cluster. **The plot displays the pairwise D' relationship between the 77 SNPs with a MAF ≥ 0.1.

Tag SNPs were selected for *IFNB1*, *IFNW1*, *ICSBP1 *and *IFNG*. However, owing to the high homology and consequent LD among the *IFNA *cluster (Figure 1), tag SNPs were chosen for the region encompassing the 13 IFNA genes, rather than for each individual gene. The tag SNP approach uses the resequencing genotype data to investigate the ability of a smaller subset of SNPs to predict the genotypes of the remainder. Predictive performance is assessed using a *R*^2 ^measure (coefficient of determination), which measures the ability to predict each known SNP genotype by linear regression on the tag SNP genotypes[10,11]. Generally we only consider SNPs with a MAF ≥ 0.05. However, as *IFNG *polymorphisms were extracted from the SeattleSNP database of 23 Caucasian individuals, we increased the MAF ≥ 0.1[23] for the analysis of this gene. Likewise, as a result of the complexity of genotyping SNPs in the *IFNA *region and the large number of SNPs with a MAF ≥ 0.05, we used a MAF ≥ 0.1 when selecting tag SNPs for the *IFNA *region. We required the subset of tags SNPs to predict the remaining SNPs with a minimum *R*^2 ^of 0.8

### DNA collections

Tag SNPs for all genes were genotyped in a family collection consisting of 472 multiplex families from the Diabetes UK Warren 1 collection and 268 multiplex families from the (USA) Human Biological Data Interchange, providing up to 1,427 parent-child trios. Ethical approval by the relevant research ethics committees was obtained for all DNA samples collected, and written informed consent was obtained from the participants.

### Genotyping

Genotyping of the tag SNPs was performed using either Taqman (ABI) or Invader (Third Wave Technologies) assays with the exception of rs35085912, a DIP, for which fluorescent primers were designed (Primer1:CGCCTCTTATGTACCCACAAA-FAM Primer2:TTTTTCTGATTGAATCTCCCATT) and size differences discriminated using an ABI3700 DNA analyser. Owing to the exceptionally high degree of sequence homology within the IFNA genes and their 3' and 5' flanking regions it was necessary to modify the standard Taqman genotyping protocol for over half the tag SNPs in this region. This was achieved through a PCR amplification of a sequence specific to the region containing the polymorphism of interest, which was subsequently used as the template for Taqman assay. An initial quality assurance pilot study was carried out on each of the SNP-specific Taqman kits before genotyping on the full T1D family set commenced. A panel of 96 T1D patients were genotyped using each respective Taqman kit. This 96 patient panel included the 32 patients used for the resequencing efforts described above for initial identification of SNPs and allele frequencies. All kits that showed 100% correlation between Taqman kit genotyping results and resequencing results were used to genotype native genomic DNA from the families (*i.e*. the standard protocol was followed). Those Taqman kits that failed to show 100% concordance with the sequencing data were either: 1) replaced by an alternative tag SNP; or, 2) reassessed through genotyping on the 96 panel after an initial nested PCR amplification step (to the isolated genomic region containing the specific SNP of interest), only those kits that showed 100% concordance with results obtained in the 96 patient DNA genotyping and 32 patient DNA sequencing panels after the nested PCR step were used for genotyping in the T1D family panel. All eight kits requiring nested PCR prior to genotyping showed 100% concordance with sequencing data from the 32 DNA resequencing set.

### Statistical analysis

Tag SNPs were analysed using a multilocus test, which essentially tests for an association between T1D and the tag SNPs due to LD with one or more causal variants[10,11]. The programs for the selection of tag SNPs and association analysis used in this paper are implemented in the *Stata *statistical system[32] and may be downloaded from David Clayton's website[33].

## Authors' contributions

GAJM and CEL participated in the design of the study, carried out gene annotation, sequencing, genotyping, data analysis and manuscript preparation. JDC participated in the design of the study, performed data analysis and participated in manuscript preparation. AV carried out gene annotation, sequencing and genotyping. FP, LG and JSH carried out genotyping. NMW coordinated data management.

BCH and ACL participated in genome informatics. PAL participated in the conception and design of the study. JAT participated in the conception, design and coordination of the study and participated in manuscript preparation. All Authors read and approved the final manuscript.

## Supplementary Material

Additional File 1 **SNPs identified in the *IFNA *region. **Novel SNPs are denoted by "ss" numbers and previously published SNPs are denoted by "rs" numbers. The gene refers to the IFNA gene in closest proximity to the SNP. Minor allele frequencies are based on the sequencing panel as listed in Table 1. *R*^2 ^values for non-typed SNPs. Tag SNPs are marked in bold. UTR, untranslated region. Click here for file

Additional File 2 **Genotyping summary for *IFNA *tag SNPs. **Genotype summary for the *IFNA *tag SNPs. Minor allele frequencies are based on parental genotypes. Number of parent-child trios obtained by population. Click here for file

Additional File 3 **SNPs identified in the *IFNB1 *region. **Novel SNPs are denoted by "ss" numbers and previously published SNPs are denoted by "rs" numbers. *R*^2 ^values for non-typed SNPs. Tag SNPs are marked in bold. UTR, untranslated region. Click here for file

Additional File 4 **Genotyping summary for *IFNB1 *tag SNPs. **Genotype summary for the *IFNB1 *tag SNPs. Minor allele frequencies are based on parental genotypes. Number of parent-child trios obtained by population. Click here for file

Additional File 5 **SNPs identified in the *IFNG *region. **Novel SNPs are denoted by "ss" numbers and previously published SNPs are denoted by "rs" numbers. *R*^2 ^values for non-typed SNPs. Tag SNPs are marked in bold. UTR, untranslated region. Click here for file

Additional File 6 **Genotyping summary for *IFNG *tag SNPs and rs2430561. **Genotype summary for the *IFNG *tag SNPs and rs2430561. Minor allele frequencies are based on parental genotypes. Number of parent-child trios obtained by population. Click here for file

Additional File 7 **SNPs identified in the *IFNW1 *region. **Novel SNPs are denoted by "ss" numbers and previously published SNPs are denoted by "rs" numbers. *R*^2 ^values for non-typed SNPs. Tag SNPs are marked in bold. UTR, untranslated region. Click here for file

Additional File 8 **Genotyping summary for *IFNW1 *tag SNPs. **Genotype summary for the *IFNW1 *tag SNPs. Minor allele frequencies are based on parental genotypes. Number of parent-child trios obtained by population. Click here for file

Additional File 9 **SNPs identified in the *ICSBP1 *region. **Novel SNPs are denoted by "ss" numbers and previously published SNPs are denoted by "rs" numbers. *R*^2 ^values for non-typed SNPs. Tag SNPs are marked in bold. UTR, untranslated region. Click here for file

Additional File 10 **Genotyping summary for *ICSBP1 *tag SNPs. **Genotype summary for the *ICSBP1 *tag SNPs. Minor allele frequencies are based on parental genotypes. Number of parent-child trios obtained by population. Click here for file
